# Infant feeding in Sweden: Socio-demographic determinants and associations with adiposity in childhood and adolescence

**DOI:** 10.1186/1746-4358-3-23

**Published:** 2008-09-16

**Authors:** Andrej M Grjibovski, Bettina Ehrenblad, Agneta Yngve

**Affiliations:** 1 Norwegian Institute of Public Health, Post Box 4404 Nydalen, N-0403 Oslo, Norway; 2 Unit for Public Health Nutrition, Department of Biosciences and Nutrition, Karolinska Institutet, SE-141 57, Huddinge, Stockholm, Sweden

## Abstract

**Background:**

Recent reviews and meta-analyses conclude that breastfeeding constitutes a small but consistent protective effect against obesity or higher values of body mass index (BMI) in children, though in some studies this effect was weakened after adjustment for potential confounders. The aim of this study was to explore the socio-demographic determinants of the duration of breastfeeding in Sweden and the associations between breastfeeding duration and adiposity in childhood and adolescence.

**Methods:**

This was a cross-sectional study of Swedish children. Height, weight and waist circumference were measured and the sum of five skin fold measurements were obtained in 1137 9- and 15-year old children. Breastfeeding data were retrospectively obtained from the medical records for 812 (71.4%) children. Multiple ordinal logistic regression was applied to study individual effects of the maternal characteristics on the duration of breastfeeding. The relationship between children's anthropometric characteristics and duration of breastfeeding was studied by multiple linear regression. Associations between the odds of being overweight or obese and the duration of breastfeeding were studied by multiple logistic regression. Both linear and logistic models were adjusted for children's age, gender, birth weight, maternal education and parental BMI in 1998 as well as maternal age and smoking status at childbirth.

**Results:**

Maternal education was positively associated with the duration of breastfeeding in both 1983 and 1989. Non-smoking mothers were more likely to breastfeed longer than smokers in 1989 (OR = 1.9, 95%CI: 1.3, 3.0). Fifteen-year old children breastfed for shorter than 2 months had 1.2 kg/m^2 ^(95%CI: 0.1, 2.4) higher BMI, 3.2 cm (95%CI: 0.2, 6.2) higher waist circumference and 10.6 mm (95%CI: 1.7, 19.6) higher sum of five skin fold measurements compared to those breastfed for 6 months or longer when adjusted for children's characteristics and maternal characteristics in 1998. Adjustment for maternal age and smoking status at childbirth weakened these associations to non-significant levels (0.9 kg/m^2^, 95%CI: -0.4, 2.1; 1.4 cm, 95%CI: -1.5, 4.4 and 5.1 mm, 95%CI: -4.0, 14.2, respectively). In the 9-year olds, the associations were less pronounced, but in the same direction. No trends between duration of breastfeeding and children's anthropometric characteristics were observed in any of the age groups.

**Conclusion:**

Maternal education and smoking were significant predictors of breastfeeding duration in Sweden in the 1980s. Associations with measures of adiposity were observed only in 15-year old children between the children with shortest and longest breastfeeding duration, which were weakened after adjustment for maternal characteristics at childbirth.

## Background

The importance of breastfeeding for both maternal and child health is widely recognised. Maternal short-term risks of not breastfeeding include increased risk of postpartum haemorrhage while long-term effects may include a higher risk of osteoporosis as well as breast and ovarian cancers [1]. Infants who are not breastfed have documented increased risk of infectious and atopic diseases and impaired psychomotor development [2]. The relationship between breastfeeding and obesity in childhood and adolescence is less clear [3]. Recent reviews and meta-analyses conclude that breastfeeding constitutes a small but consistent protective effect against obesity or higher values of body mass index (BMI) in children [4-7], though in some studies this effect was weakened after adjustment for potential confounders [7-12]. Studies which estimate the effects of exclusive breastfeeding on childhood obesity also suggest a protective effect [11], although this effect was not found in some ethnic groups or in smokers [12]. Considerable heterogeneity in definitions and duration of breastfeeding, criteria for overweight and obesity, age of children when the measurements were taken, and confounding factors used for adjustment, could contribute to the inconsistencies in the results of the studies. Selective reporting and publication bias could also influence the available evidence.

Most of the studies on the effect of infant feeding on overweight and obesity have used BMI as the main outcome. However, it is body adiposity, rather than weight in relation to height, that is related to the obesity-associated diseases. Skin fold measurements provide better assessment of adiposity than BMI [13]. Studies that used skin folds as a measure of obesity in relation to breastfeeding were small and yielded inconsistent results [12,14,15]. Moreover, Burdette et al found no relationship between the duration of breastfeeding and adiposity in 5-year old children using dual-energy X-ray absorptiometry (DXA) and suggested that the effect of breastfeeding on obesity may vary depending on the measure of adiposity used [16]. Thus, there is a need for further research on the role of breastfeeding as protection against childhood and adolescent obesity using other measures of adiposity than BMI.

Sweden is a country with almost universal initiation and long duration of breastfeeding compared to other industrialised nations [17]. The initiation and duration of breastfeeding depend on a number of determinants, which could be summarised in five groups: socio-demographic, psychosocial, biomedical and health care-related factors, community attributes, and public policy [18]. However, the importance of these factors varies across countries and over time [19]. We identified only one study specifically addressing socio-economic determinants of breastfeeding in Sweden [20]. However, this study reports the determinants of exclusive breastfeeding only. Other studies have reported that duration of any breastfeeding in Sweden was positively associated with maternal education, inversely with smoking and not associated with maternal age [21,22].

Our study aims to estimate the effects of selected socio-demographic factors on the duration of breastfeeding in Sweden and to study the associations between the duration of breastfeeding and adiposity in childhood and adolescence using data from a Swedish study of children and adolescents and from medical records.

## Methods

This study was performed as an extension of a cross-sectional survey performed in 1998. The sample included 1137 children born in 1983 and 1989 from randomly selected schools in two areas in central Sweden, namely, Södertörn (Southern Stockholm) and Örebro [23].

### Data collection

The data on infant feeding status of the participants at 2 weeks, 2, 4, and 6 months as well as their birth weight were retrospectively collected from the paediatric files at local health care centres one year after the survey. These time points were selected to ensure comparability of our data with the national statistics [17]. The term breastfeeding here refers to any breastfeeding at the specified time points.

Data on all other studied factors were collected in 1998. Body weight was measured to the nearest 0.1 kg on a SECA digital beam balance, calibrated after transportation. Height was measured to the nearest 5 mm, using a Harpenden transportable stadiometer. The subjects were dressed in light underwear and no shoes. Overweight (including obesity) was defined using the International Obesity Task Force (IOTF) cut-off criteria [24]. Later in the text, the term overweight is used to cover overweight including obesity. Biceps, triceps, subscapular, suprailiac, and calf skin fold thickness was measured using Harpenden calipers according to Lohman [25]. The sum of all five skin fold measurements (in mm) was calculated. Waist circumference (in cm) was measured using a metal anthropometric tape midway between the lower rib margin and the iliac crest, at the end of gentle expiration. Pubertal maturation was assessed according to Tanner [26]. Further details on sampling procedure, participation rates and data collection are presented elsewhere [23].

The data on parental height, weight and maternal education in 1998 as well as on maternal age and smoking status at the time of childbirth were obtained from the parents' questionnaire. Education was categorised as basic (9 years), high school (11–12 years) and university education. By smoking status at childbirth, women were categorized as non-smokers or smokers using the reported data on age at initiation and cessation of smoking. Maternal age at childbirth was divided in to three categories: < 25 years, 25 – 34, and ≥ 35 years. Maternal and paternal BMI were calculated using the self-reported height and weight data.

### Data analysis

Differences between breastfeeding rates in 1983 and 1989 were compared by log-rank tests in Kaplan-Meier analysis. Individual effects of each of the maternal socio-demographic characteristics on the duration of breastfeeding were assessed using multiple ordinal logistic regression analysis given the ordinal nature of the dependent variable. Odds ratios (OR) with 95% confidence intervals (CI) were calculated. OR above one suggests higher odds for longer breastfeeding. Maternal basic education, age of 35 years or older, smoking during pregnancy and female gender of the baby were used as reference categories.

Given that very few children were never breastfed, four categories of the duration of breastfeeding were used in all other analyses: < 2 months, 2 – 3 months, 4 – 5 months and ≥ 6 months. Independent effects of breastfeeding on the studied anthropometric parameters were assessed using multiple linear regression. Logistic regression was used to study the associations between the duration of breastfeeding and overweight at 9 and 15 years. Child age, gender and Tanner stage of pubertal maturation, maternal education, maternal and paternal BMI at the time of the study were used as covariates in the first model. In the second model, additional adjustment for maternal age and smoking status at childbirth was made. Tests for trends were performed by introducing the duration of breastfeeding in the models as a continuous variable. All analyses were performed using SPSS, version 13.0. (SPSS Inc., Chicago, IL). Analyses were performed separately in 9- and 15-year old children.

Approval for the cross-sectional survey and for the breastfeeding data collection and analysis were obtained from the local Medical Ethics' committees in Stockholm and Örebro.

## Results

The data on breastfeeding were available for 812 (71.4%) of the 1137 subjects enrolled in the study. No differences by education, age, smoking status and gender of the children were found between mothers with missing and available data on breastfeeding within the year of childbirth except for the slightly higher proportion of younger mothers in the group with missing data on breastfeeding in those who gave birth in 1983 (p = 0.04). Maternal and children's characteristics for the participants with available data on breastfeeding are presented in Table 1.

**Table 1:** Characteristics of mothers and their children with available data on breastfeeding by the children's birth year

	**1983** **(n = 394)**	**1989** **(n = 418)**
**Maternal characteristics**		
Age at childbirth, n (%)		
<25	85 (21.6)	60 (14.4)
25–34	212 (53.8)	217 (51.9)
35+	47 (11.9)	43 (10.3)
Unknown	50 (12.7)	98 (23.4)
Education, n (%)		
Basic	76 (19.3)	60 (14.4)
High school	139 (35.3)	222 (53.1)
University	160 (40.6)	120 (28.7)
Unknown	19 (4.8)	16 (3.8)
Smoking at childbirth, n (%)		
Non-smokers	201 (51.0)	182 (43.5)
Smokers	145 (36.8)	140 (33.5)
Unknown	48 (12.2)	96 (23.0)
**Parents' BMI**		
Current maternal BMI, M (SD)	24.1 (3.9)	23.8 (3.9)
Current paternal BMI, M (SD)	25.7 (3.0)	25.6 (3.2)
**Children's characteristics**		
Gender, n (%)		
Boys	176 (44.7)	208 (49.8)
Girls	218 (55.3)	210 (50.2)
Age, M (SD)	15.5 (0.4)	9.6 (0.3)
Overweight, n (%)	43 (10.9)	64 (15.3)
BMI, kg/m^2^, M (SD)	20.7 (2.6)	17.4 (2.5)
Waist circumference, cm, M (SD)	71.2 (6.8)	60.8 (6.4)
Sum of five skin fold measurements, mm, M (SD)	53.0 (21.2)	46.2 (21.2)

### Breastfeeding rates and correlates of breastfeeding duration

**Figure 1:**
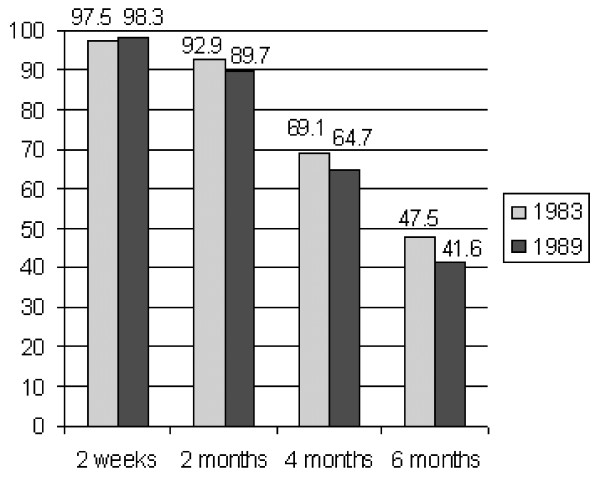
Proportion of children (%) breastfed at 2 weeks, 2, 4 and 6 months of age.

The rates of breastfeeding at 2 weeks, 2, 4 and 6 months are presented in Figure 1. Women who gave birth in 1983 were more likely to breastfeed longer than those who gave birth in 1989 (p = 0.05). Maternal education was positively associated with longer duration of breastfeeding in both 1983 and 1989 (Table 2). The variations were more pronounced in 1983. Non-smokers were more likely to breastfeed longer than smokers in 1989, according to the recall data on smoking. The associations between breastfeeding duration and smoking in 1983 were in the same direction. Boys were breastfed for a shorter time than girls in 1989, while no differences were observed in 1983.

**Table 2:** Adjusted odds ratios* (OR) for longer breastfeeding by maternal characteristics

	**1983**	**1989**
	**OR (95% CI)**	**OR (95% CI)**

**Maternal education**		
University	4.73 (2.69, 8.30)	2.15 (1.11, 4.15)
High school	2.73 (1.70, 4.41)	1.26 (0.78, 2.05)
Basic	1.00 (Reference)	1.00 (Reference)
**Maternal smoking**		
No	1.49 (0.98, 2.26)	1.94 (1.27, 2.97)
Yes	1.00 (Reference)	1.00 (Reference)
**Maternal age**		
< 25	0.92 (0.45, 1.90)	1.71 (0.81, 3.63)
25–34	1.16 (0.61, 2.18)	1.30 (0.69, 2.45)
≥ 35	1.00 (Reference)	1.00 (Reference)
**Gender of the baby**		
Boy	1.05 (0.70, 1.57)	0.66 (0.44, 0.99)
Girl	1.00 (Reference)	1.00 (Reference)

*Adjusted for all variables in the table.

### Duration of breastfeeding and measures of adiposity

Both 9- and 15-year old children from the group with the shortest breastfeeding duration had the highest values of BMI, waist circumference and sum of five skin fold measurements (Table 3). In crude analyses, 15-year old children who were breastfed for less than 2 months had higher BMI and waist circumferences than their counterparts who were breastfed for 6 or more months. Moreover, we observed inverse trends between duration of breastfeeding and both BMI and waist circumference. The differences in sum of skin fold measurements were even more pronounced, but did not reach the level of statistical significance due to greater variability. The pattern was similar for the 9-year old children, although less pronounced and not reaching the level of statistical significance (Table 4).

**Table 3:** Children's anthropometric characteristics (mean values, with standard deviation in brackets) by duration of breastfeeding

	**15-year old children**			**9-year old children**		
**Duration of breastfeeding**	**BMI, kg/m^2^**	**Waist circumference, cm**	**Sum of five skin folds, mm**	**BMI, kg/m^2^**	**Waist circumference, cm**	**Sum of five skin folds, mm**

< 2 months	21.8 (3.8)	75.3 (11.2)	59.8 (28.2)	17.7 (3.4)	62.2 (9.1)	47.9 (29.8)
2–3 months	20.6 (2.6)	70.3 (6.2)	53.1 (24.0)	17.3 (2.3)	60.9 (5.8)	45.1 (19.2)
4–5 months	20.6 (2.4)	71.6 (5.8)	53.5 (21.9)	17.3 (2.4)	60.2 (6.3)	46.8 (22.9)
≥ 6 months	20.7 (2.4)	70.8 (6.5)	51.7 (22.5)	17.3 (2.5)	60.8 (6.2)	46.0 (19.2)

**Table 4:** Crude and adjusted differences with 95% confidence intervals (CI) in BMI, waist circumference and sum of five skin folds by duration of breastfeeding.

	**15-years old children**			**9-years old children**		
	**BMI, kg/m^2^**	**Waist circumference, cm**	**Sum of five skin folds, mm**	**BMI, kg/m^2^**	**Waist circumference, cm**	**Sum of five skin folds, mm**

Crude						
< 2 months	1.2 (0.1, 2.2)	4.5 (1.7, 7.4)	8.1 (-1.4, 18.1)	0.4 (-0.5, 1.2)	1.3 (-0.9, 3.4)	1.5 (-5.7, 8.7)
2–3 months	-0.1 (-0.8, 0.5)	-0.5 (-2.3, 1.3)	1.4 (-4.7, 7.6)	0 (-0.6, 0.6)	0.1 (-1.5, 1.7)	-0.8 (-6.1, 4.4)
4–5 months	-0.1 (-0.8, 0.6)	0.9 (-1.0, 2.7)	1.8 (-4.7, 8.4)	0 (-0.6, 0.6)	-0.5 (-2.1, 1.1)	0.6 (-4.7, 6.0)
6+ months	Reference	Reference	Reference	Reference	Reference	Reference
p-value for trend	0.033	0.003	0.129	0.485	0.242	0.864
						
Adjusted						
< 2 months	1.2 (0.1, 2.4)	3.2 (0.2, 6.2)	10.6 (1.7, 19.6)	0.5 (-0.4, 1.5)	1.8 (-0.5, 4.1)	3.2 (-4.7, 11.0)
2–3 months	-0.4 (-1.1, 0.3)	-0.8 (-2.6, 1.1)	-6.1 (-11.8, -0.4)	-0.1 (-0.7, 0.6)	0.2 (-1.6, 2.0)	-1.9 (-7.6, 3.9)
4–5 months	-0.2 (-0.9, 0.5)	0.5 (-1.3, 2.4)	0.7 (-4.9, 6.2)	0.1 (-0.6, 0.8)	0.1 (-1.7, 1.8)	1.4 (-4.3, 6.9)
6+ months	Reference	Reference	Reference	Reference	Reference	Reference
p-value for trend	0.725	0.439	0.973	0.485	0.242	0.864

*Adjusted for children's age, gender, pubertal maturation, birthweight, maternal education, maternal and paternal BMI

Adjustment for children's characteristics and parental data at the time of the study only marginally changed the findings. Fifteen-year old children who were breastfed for shorter than 2 months had higher values for BMI, waist circumference and sum of five skin fold measurements by 1.2 kg/m^2^, 3.2 cm, and 10.6 mm, respectively, compared to children who were breastfed for 6 months or longer. Children from the same age group who were breastfed for 2–3 months had 6.1 mm lower sum of skin fold measurements than the reference group. Similar, but non-significant associations were observed in 9-year old children.

Adjustment for maternal age and smoking status at childbirth weakened all previously described associations to non-significant levels. Fifteen-year old children who were breastfed for shorter than 2 months continued to have higher values for BMI, waist circumference and sum of skin fold measurements compared to the reference group, although the differences were reduced by about one third for BMI and by half for other measures. The only significant association was observed for 15-year old children who were breastfed for 2–3 months, who had 6.9 cm lower sum of skin fold measurements than the reference group. No trends between the duration breastfeeding and children's anthropometric indices were observed (Table 4).

### Duration of breastfeeding and overweight

Altogether, 10.9% (95%CI: 8.2, 14.4) of 15-year old and 15.3% (95%CI: 12.2, 19.1) of 9-year old children were overweight. In the group with the shortest duration of breastfeeding, the proportion of overweight was 25.0% in the 15-year old and 20.5% in the 9-year old children while in the group with the longest duration of breastfeeding, the corresponding proportions were 9.7% and 14.6% (Table 5). In crude analysis, 15-year old children who were breastfed for less than 2 months had considerably higher odds for being overweight (OR = 3.1, 95%CI: 1.2, 8.3) than those who were breastfed for 6 months or longer.

**Table 5:** Prevalence of overweight, crude and adjusted odds ratios (OR) with 95% confidence intervals (CI) for overweight by duration of breastfeeding

**Duration of breastfeeding**	**% Overweight** **(95% CI)**	**Crude OR** **(95% CI)**	Adjusted OR* (95% CI)	**Adjusted OR^†^** **(95% CI)**
**15-year-olds**				
< 2 months	25.0 (12.7, 43.5)	3.1 (1.2, 8.3)	2.2 (0.6, 7.7)	1.0 (0.2, 5.6)
2–3 months	10.6 (5.9, 18.5)	1.1 (0.5, 2.5)	0.6 (0.2, 1.7)	0.6 (0.2, 2.2)
4–5 months	9.4 (4.9, 17.5)	1.0 (0.4, 2.3)	0.9 (0.4, 2.3)	1.0 (0.3, 3.3)
≥ 6 months	9.7 (6.2, 14.8)	Reference	Reference	Reference
p-value^‡^		0.102	0.359	0.881
p-value for trend^§^		0.107	0.966	0.688
**9-year-olds**				
< 2 months	20.9 (11.5, 35.3)	1.5 (0.7, 3.5)	1.5 (0.6, 3.9)	1.2 (0.3, 4.6)
2–3 months	12.5 (7.5, 20.2)	0.8 (0.4, 1.7)	0.7 (0.3, 1.6)	0.6 (0.2, 1.7)
4–5 months	17.7 (11.4, 26.6)	1.2 (0.6, 2.5)	1.2 (0.6, 2.6)	0.5 (0.1, 1.5)
≥ 6 months	14.5 (10.1, 20.6)	Reference	Reference	Reference
p-value^‡^		0.538	0.501	0.430
p-value for trend^§^		0.673	0.977	0.745

*Adjusted for children's age, gender, pubertal maturation, birth weight, maternal education, maternal and paternal BMI

† + maternal age and smoking status at childbirth

^‡^Performed by Pearson's χ^2 ^test in univariate analysis and Wald test in logistic regression

^§^Performed by introducing breastfeeding categories in the model as a continuous variable

Adjustment for children's data, maternal education and parental BMI at the time of the study weakened this association to OR = 2.2 (95%CI: 0.6, 7.7). Further adjustment for maternal age and smoking status at childbirth further reduced this odds ratio to OR = 1.0 (95%CI: 0.2, 5.6). Other results in this age group remained largely unchanged.

In 9-year old children, the odds of being overweight were 50% higher for the group with the shortest duration of breastfeeding in crude analysis, but the CI were wide and included 1. Adjustment for other factors further weakened this association. Moreover, children who were breastfed for 2–3 and 4–5 months tended to have lower odds of being overweight compared to the reference group.

## Discussion

The results suggest that in spite of the egalitarian nature of the Swedish society, there were considerable variations in the duration of breastfeeding by maternal education and smoking status during the 1980s. Moreover, we found that the children who were breastfed for shorter than 2 months had higher values for BMI, waist circumference and sum of five skin fold measurements at 15 year compared to their counterparts who were breastfed for 6 months or longer independently of birth weight, age, gender, parental BMI and maternal education. However, adjustment for maternal age and smoking at childbirth substantially reduced these associations. No dose-response relationship between the duration of breastfeeding and any of the studied measures of adiposity were found. We also observed that 15-year old children who were breastfed for shorter than 2 months had 3 times higher odds of being overweight than their counterparts breastfed for more than 6 months in a crude analysis, but this association disappeared after adjustment for other factors. The associations for 9-year olds were in the same direction, although they were less pronounced and did not reach the level of statistical significance.

The representativeness of the sample in relation to the general population in the studied areas [23], prospective registration of the breastfeeding status by health professionals, objective measurements of children's and adolescents' anthropometric characteristics are the main strengths of the study. No differences in socio-demographic factors between mothers with available and missing data on breastfeeding suggest that our estimates of breastfeeding prevalence are likely to be valid for the studied areas. The national breastfeeding statistics for 1989 reported the prevalence of any breastfeeding to be 97.7%, 85.8%, 67.7%, and 49.8% at 2 weeks, 2, 4, and 6 months, respectively, which is slightly above our estimates at 4 and 6 months (98.3%, 89.7%, 64.7%, and 41.6%). This is not surprising given that the studied areas were consistently reported to have breastfeeding rates below the national average [17] in previous national surveys.

Socio-demographic factors are important determinants of breastfeeding [18,19]. Our findings on variations in breastfeeding duration by maternal education are in line with the results of other studies carried out in Sweden, which also found that low education was associated with shorter duration of breastfeeding [20,21]. Maternal education in this study is defined as the highest attained education, not education at childbirth. This might be a better indicator reflecting the educational potential of the mother, her ambitions and the possibility of studying after childbirth. The underlying mechanisms of the associations between education and duration of breastfeeding are unclear; though we hypothesize that better educational potential of the mothers might lead to better compliance to the recommendations provided by the health care staff regarding breastfeeding. Although education is considered to be the most informative indicator of women's socioeconomic status in Sweden, there is still a chance for residual confounding by other factors. One study from Sweden reported no association between maternal age and duration of breastfeeding, which is in line with our results [22]. There is convincing evidence that smoking is associated with shorter duration of breastfeeding [19-22,27,28]. We obtained similar associations in the present study.

The self-reported nature of the maternal data may threaten the validity of the study, although this is unlikely given that previous studies from Sweden reported acceptable validity of self-reported data on obesity in women [29] and smoking during pregnancy [30]. Moreover, the prevalence of smoking in pregnancy in Sweden was about 30% during the 1980s with some tendency to decrease during the decade [31]. Our data show the prevalence of smoking to be 37% and 34% in 1983 and 1989, respectively, suggesting that underestimation is unlikely.

Similarly to most studies, our findings suggest that infants who are breastfed for shorter than 2 months have higher BMI, larger waistline and thicker skin fold measurements at 15 year than their counterparts breastfed for 6 months or more [3-8,32,33], but only before adjustment for maternal age and smoking at the time of childbirth. Several other studies also report attenuation of crude associations between measures of adiposity and breastfeeding after adjustment for confounders [6,8-11]. No dose-response effect of the duration of breastfeeding on any of the studied measures of adiposity in either 9- or 15-year old children was observed. Interestingly, after adjustment, the lowest values for all measures of adiposity in both 15- and 9-year old children were observed in the group breastfed for 2–3 months reaching the level of statistical significance for sum of skin fold measurements in 15-year-olds.

Similarly to what was observed for the continuous measures of adiposity, crude associations between breastfeeding and overweight were substantially reduced after adjustment for maternal age and smoking status at childbirth. Moreover, adjustment for other studied characteristics resulted in lower odds ratios for overweight for 15-year old children breastfed for 2–3 months (OR = 0.7, 95%CI: 0.2, 2.2) and for 9 year old children breastfed for both 2–3 (OR = 0.6, 95%CI: 0.2, 1.7) and 4–5 months (OR = 0.5, 95%CI: 0.1, 1.5). Although the results were not statistically significant, they are congruent with the results of the analyses performed on BMI, waist circumference and sum of skin fold measurements. Victora et al also found 50% reduction in obesity in children breastfed for 3–5 months making it tempting to relate all these findings to the "critical window" theory of development [9]. However, wide confidence intervals for all associations in our study do not allow any conclusions.

The results of the study should be interpreted with caution, taking into account the limitations of the study. Firstly, the small sample size leads to insufficient statistical power to detect small differences in adiposity measures and odds for overweight by breastfeeding status. Secondly, the use of multiple linear regression while analysing slightly skewed data on BMI, waist circumference and skin fold measurements might be criticized. However, the distributions of the residuals were approximately normal and other assumptions were not violated. Moreover, repeated analyses on logarithmically transformed data yielded similar results.

Although our results do not contradict conclusions of large meta-analyses and systematic reviews on the effect of breastfeeding on obesity in childhood and adolescents, less pronounced differences between the groups of breastfeeding duration may partly be explained by the choice of the reference group. While in often cited reviews [4,6] and meta-analyses the reference group included formula fed infants, in our study, the reference group was different. Contrary to many other studies, the proportion of never breastfed infants in our setting was very small. Only 2.5% of 15-year old and 1.8% of 9-year old children in our sample were not breastfeed at 2 weeks (Figure 1). Thus, our reference group included children, most of who were breastfed, but for shorter period than two months. Moreover, given that initiation rates and average duration of breastfeeding in Sweden are greater and the prevalence of childhood overweight and obesity is lower than in many other European countries and the USA, the associations between breastfeeding duration and later adiposity may be less visible in Sweden than in countries where breastfeeding is less popular and childhood obesity is more prevalent.

## Conclusion

In conclusion, we observed considerable social variations in breastfeeding duration in Sweden during the 1980s, and found that 15-year old adolescents, who were breastfed for less than 2 months, had a higher BMI, larger waist circumference and sum of five skin fold measurements compared to their counterparts who were breastfed for 6 months or longer, although the associations were no longer significant after adjustment for maternal age and smoking status at childbirth. Similar, but less pronounced associations were observed in 9-year old children. We could not replicate previously reported dose-response associations between the duration of breastfeeding and measures of adiposity or prevalence of overweight. Thus the results are generally in the expected direction, but inconclusive and should be interpreted and generalized with caution, given the limitations of the study design and almost universal initiation of breastfeeding in Sweden.

## Competing interests

The authors declare that they have no competing interests.

## Authors' contributions

AMG drafted the manuscript and performed the statistical analysis. BE helped to design the study, collected the breastfeeding data from the medical records and entered these into the data base. AY conceived the study, participated in its design and coordination and drafted the manuscript. All authors approved the final manuscript.
